# Exploring Canadian Children’s Social Media Use, Digital Literacy, and Quality of Life: Pilot Cross-sectional Survey Study

**DOI:** 10.2196/18771

**Published:** 2021-05-26

**Authors:** Lorie Donelle, Danica Facca, Shauna Burke, Bradley Hiebert, Emma Bender, Stephen Ling

**Affiliations:** 1 Arthur Labatt Family School of Nursing The University of Western Ontario London, ON Canada; 2 School of Health Studies The University of Western Ontario London, ON Canada; 3 Schulich School of Medicine & Dentistry The University of Western Ontario London, ON Canada

**Keywords:** child, children, internet, social media, digital literacy, digital inclusion, quality of life, mobile phone

## Abstract

**Background:**

Understanding social media use and digital literacy among young Canadian children is an increasing area of concern, given the importance of digital inclusion for full and informed participation in evolving educational, civic, corporate, social, and economic spaces.

**Objective:**

The aim of this study was to explore internet and social media knowledge as well as social media use among Canadian children aged between 6 and 10 years.

**Methods:**

We conducted interview surveys with 42 children aged between 6 and 10 years who participated in an after-school health promotion program in an urban community in Southwestern Ontario to understand their digital literacy skills and social media use. The data were analyzed using both quantitative and qualitative methods.

**Results:**

Of the 42 children who participated in this study, 24 (57%) reported that they used social media, specifically YouTube (19/24, 79% reported use), Snapchat (16/24, 67% reported use), and Facebook (8/24, 33% reported use). While using social media, children reported sharing personal information, including videos or pictures of themselves (12/24, 50%), videos or pictures of others (8/24, 33%), and their birthday (12/24, 50%), whereas only one-third (9/24, 38%) of the children believed that only close family and friends had access to the content they shared. When reporting on the quality of life in the context of using social media, most (17/24, 71%) children never felt sad, half (12/24, 50%) never had difficulty making new friends, and nearly one-third (7/24, 30%) indicated that they never had difficulty wanting to play outside.

**Conclusions:**

Owing to the rapidly evolving uptake and use of social media among young Canadians, the implementation of childhood digital health literacy education is vital to best support digital inclusion and well-being in Canada. The findings of our study highlight the need for future research to understand where children receive their digital literacy knowledge from and whether this knowledge is gained through self-directed social media use or observation from other actors, such as parents, siblings, or friends.

## Introduction

### Background

The infiltration of digital devices across the age continuum supports the need for widespread attention to digital literacy. In general, digital literacy refers to a person’s knowledge and skills in using digital devices to perform a variety of tasks, including sharing knowledge and information; communicating with others; participating in web-based communities, such as social media platforms; creating digital content; and analyzing digital content from a critical lens [1,2]. The practical value of digital literacy provides the necessary knowledge to optimize the social benefits and opportunities afforded by digital devices. Fostering digital literacy skills is crucial for ensuring digital inclusion and resolving inequities related to the digital divide [3]. Digital inclusion refers to mending the digital divide in terms of enhancing equitable access to digital devices and the internet so that individuals can meaningfully participate in all sectors of a knowledge- and information-driven society [3,4]. Considering North America’s information and data-powered infrastructure, digital literacy skills are, and will continue to be, a crucial component of full and informed participation within evolving educational, civic, corporate, social, and economic spaces [5-7].

Digital literacy and the extent to which someone can effectively use digital devices to navigate and participate in sectors across contemporary society have implications for perceived quality of life, in other words, factors that contribute to life satisfaction among users [8,9]. From a subjective perspective, perceived quality of life is influenced by personality factors and an individual’s perception of their position in life in relation to their goals and concerns, including their sense of self-worth, personal development, and feelings of isolation. From an objective perspective, perceived quality of life is influenced by environmental factors, including one’s family, job, community, and standard of living. It has been noted in the literature that satisfaction with one’s standard of living tends to influence subjective well-being, which is important to keep in mind when considering how advancements in technology and internet connectivity have become material features that contribute to the makeup of one’s standard of living [8]. Advancements in technology, internet connectivity, and social media have fundamentally reshaped the way we work, learn, communicate with others, and cultivate our identities; thus, understanding the relationship between perceived quality of life and technology use is a necessary area of research inquiry. For example, researchers have begun to explore the effects of social media use on life satisfaction among preadolescent and adolescent populations in areas related to social (dis)connectedness, relationship conflict, emotional well-being, sleep quality, physical activity, and school performance [10-12].

In North America, for most American adolescents and young adults, being connected to others through digital devices means engagement with social media platforms, such as Facebook and Twitter [12]. Given the ample uptake of digital devices among American youth, it is not surprising that evidence from an American nationwide survey that asked parents about their children’s social media use found that 72% of children aged ≤8 years had used digital devices for playing games, watching videos, or using apps, whereas 17% of children used mobile digital devices at least once or more on a daily basis [12]. The investigators also reported that 38% of all children aged ≤2 years, 80% of children aged between 2 and 4 years, and 83% of children aged between 5 and 8 years had used a mobile digital device [13].

Recent research also notes that the frequency of social media use among older children, aged 8-11 years, and adolescents, aged 13-17 years, increases with age [14,15]. In particular, patterns of social media use among older children, aged 9-12 years, are seen to be rapidly approaching those of adolescent users [15]. For example, more than one-third of European children aged 9-12 years claimed that they had their own profile for at least one social media site [7]. Research also reports that the types of social media platforms that children and adolescents use evolve with age [15,16]. For example, YouTube was identified as the most popular social media platform for American children, followed by Moshi Monsters and Club Penguin, whereas Facebook, Instagram, and Twitter were identified as the most popular social media platforms for American adolescents [16]. For Canadian adolescents aged 13-17 years, playing web-based games, streaming music or television shows, and connecting with friends were the most common social media activities [17].

Among European children aged 9-12 years, more than one-fourth reported having their social media profile *set to public* [7]. Although European children aged 9-12 years had fewer social media profiles overall than children aged 13-16 years, they were still more likely to have their profiles set to public [7]. Research shows that children who have their social media profiles set to public are twice as likely to also post personal information than those who have their profile *set to private* or *set to partially private* [7]. It may be that children who have their profile set to public are merely unaware of how to change their privacy settings on a particular social media site [7]. As some researchers note, understanding children’s and adolescents’ awareness and use of the privacy settings on social media is essential for identity formation and maturation, as the internet may offer them a space where they can evade and control adult surveillance [16].

In Canada, between 2009 and 2014, 1.1 million adolescents and young adults aged 15-29 years reported experiences of cyberbullying or cyberstalking [18]. Cyberbullying is a global issue [19], and researchers note that using customized privacy settings and blocking features on social media is a strategic way to protect oneself from such harm [20]. When it comes to examining social relationships, conflict, cyberbullying, and personal safety, most research to date has focused only on adolescents and older children, aged ≥12 years, with little insight into younger children [21].

Although evidence shows that older children, preadolescents, and adolescents are engaging with social media alike and suggests that social media is a vital means of communication and reason for their digital device use in North America, there is limited research seeking to understand the rapidly evolving uptake and use of social media among children aged ≤12 years, which creates a significant gap in knowledge concerning their digital literacy [15,19,22,23]. In particular, research concerning social media use and digital literacy among young Canadian children is lacking. Although Canadian researchers continue to provide growing insight into the uptake and nature of digital device use among children aged ≥12 years, there is limited research on the nature of social media use and digital literacy among younger children. Research focused on this population segment can explore children’s experiences of information sharing, personal safety, cyberbullying, and learning patterns to better understand their concerns, needs, and capabilities associated with digital literacy.

It is important for researchers, educators, and parents to know how young children understand privacy in relation to social media use, given the rise of the privacy paradox: when device users claim to care about their internet privacy but act in ways that contradict this concern. For example, a device user may claim that protecting their personal information is important to them, but then freely give this personal information in exchange for services or convenience. Understanding young children’s views on privacy can provide important insights into their behaviors and the factors that influence their rationale for the choices they make when using social media [24]. By seeking a more thorough understanding of digital inclusion as it relates to younger children, researchers, educators, and policy makers can better support Canadian children to enhance their quality of life within the digital age.

### Objectives

This pilot study drew on childhood research theories that privilege children’s perspectives on their social situations, such as their participation and safety in digital spaces, by recognizing their agency and competence on matters that affect them [25-27]. As recent evidence highlights the increased importance of internet connectivity in the everyday lives of North American children and points toward an increased trajectory of use [13,22], it is vital for researchers to learn from children and work directly with them to understand their experiences. To best recognize and respect children as social actors, this pilot study focused only on young children’s self-reported digital literacy and social media use. The research questions that informed this study were as follows:

1. What knowledge of the internet and social media do Canadian children aged 6-10 years demonstrate?
2. What types of digital devices and social media platforms do Canadian children aged 6-10 years use, and how often do they use them?
3. How do Canadian children aged 6-10 years navigate personal safety and information sharing when using social media?

This pilot study aims to identify Canadian children’s knowledge of the internet and social media, the types and frequency of social media platforms they use, the types of personal information they share on social media, who has access to the personal information they share on social media, who watches them while they are on social media, who helps them download social media apps, and how they feel about their quality of life in the context of their social media use. This paper documents the findings from a survey administered through one-on-one interviews conducted among primary school-aged children in Southwestern Ontario.

## Methods

### Study Design

The pilot study used a nonexperimental cross-sectional survey design that focused on children aged between 6 and 10 years who participated in an after-school health promotion program in an urban community in Southwestern Ontario. The goal of the community-based health promotion program was to foster healthy lifestyle knowledge and skills through play. The after-school health promotion program was offered once a week over the course of 8 weeks and took place in a multipurpose room (eg, gym) within several elementary schools and public housing communities in less affluent areas of the community. In this study, social media was defined as “a set of web applications that enable production, aggregation, sharing, and remixing of content from multiple sources by mass, networked participants” [23]. The nonmedical research ethics board at Western University approved the study (file #107798).

### Participants

Eligible participants were recruited from those enrolled in the community-based after-school health promotion program and were included in the study if they were children aged 6-10 years, enrolled in primary school grades 1-3, and English speaking. Convenience sampling was used to recruit participants by sending home a letter of information about the research, a parental consent form, and a child assent form with the children. Inclusion in the study required both a signed parental consent form and a child assent form.

### Data Collection

Researchers conducted one-on-one interviews with each child lasting approximately 15 minutes in length during the after-school program’s regularly scheduled hours. Interviews were completed in a location away from the after-school program activities to safeguard the children’s confidentiality. Demographic data such as the child’s age, grade, and gender were also collected at this time. A series of closed- and open-ended questions were constructed to explore children’s digital literacy in terms of their knowledge of the internet and social media. Questions about the children’s use of social media captured whether others (eg, parents, siblings, and teachers) facilitated their use of social media (eg, who helped to download apps or access information) and monitored their activity. Questions also asked how often the children used social media, shared personal information, and implemented privacy settings.

A modified version of the Pediatric Quality of Life Inventory (PedsQL) version 4.0 Child Report [28] was used to explore children’s perceived quality of life in relation to their social media use. The original PedsQL child report questionnaire [28] consisted of 23 items encompassing four dimensions: physical functioning (eight items), emotional functioning (five items), social functioning (five items), and school functioning (five items) and was formatted using a Likert-type scale with responses ranging from 0 (eg, “it is never a problem”) to 4 (eg, “it is almost always a problem”). This study’s modified version consisted of 18 items: physical functioning (four items), emotional functioning (five items), social functioning (four items), and school functioning (five items). Researchers reverse scored the items using the PedsQL version 4.0 Generic Core Scales [29].

### Data Analysis

The data were organized and analyzed using IBM SPSS Statistics [30]. Descriptive analysis of the data was conducted. For categorical variables such as participants’ gender, grade, and types of digital devices and social media participants used, proportions and frequency counts were calculated.

## Results

### Participants

A total of 42 children were recruited to participate in this study. Overall, 50% (21/42) of the children were identified as male and 50% (21/42) of the children were identified as female. The average age of the children was 7.5 years (SD 0.943). The age range of the children was 6-10 years.

### Internet Knowledge

When asked “Do you know what the internet is?” 79% (33/42) of children described the symbols that represent connecting to the internet, including “the symbol with the E” (female participant, age 7 years), “google chrome” (male participant, age 7 years), or “a compass” (male participant, age 7 years). Others described key aspects of digital literacy stating that “you have to be careful” (female participant, age 9 years) while on the internet because it can be “full of strangers” (female participant, age 7 years) inappropriate pictures and a space both “safe and unsafe” (male participant, age 9 years). Furthermore, several of the children noted the functional role of the internet as a bridge between computer hardware and software, as they described it as what “makes the phone and iPad go” (female participant, age 8 years) while also acknowledging that “without it you can’t do much or download stuff” (male participant, age 8 years). The children also described the internet as an educational space “for reading” (male participant, age 7 years), “for homework” (male participant, age 7 years), to “search stuff” (male participant, age 8 years), and to “explore stuff, discover and talk” (male participant, age 8 years). Most predominantly, however, the children described the internet as a source of entertainment, as they considered it fundamental to access “fun games and cool things” (female participant, age 8 years), “watch YouTube” (male participant, age 7 years), or “make videos” (female participant, age 7 years).

### Social Media Knowledge, Type, and Frequency of Use

When asked “Do you know what social media is?” 54% (22/42) of children described social media in reference to the term itself or by a proprietary name such as Facebook or Snapchat. Overall, 57% (24/42) of children reported that they used social media. In terms of frequency, 46% (11/24) of children reported that they always used social media. The predominant reason among children who did not report that they used social media was that their parents did not let them use social media. Regarding the use of specific social media platforms, 79% (19/24) of children reported that they used YouTube; 67% (16/24) of children reported that they used Snapchat; and 33% (8/24) of children reported that they used Facebook, Musically, or other unspecified sites. Table 1 presents the types of social media reported by the children and the number of children who reported using it.

**Table 1:** Children’s reported social media use (n=24).

Type of social media	Children who reported use, n (%)
YouTube	19 (79)
Snapchat	16 (67)
Facebook	8 (33)
Other	8 (33)
Musically	8 (33)
Skype	7 (29)
Instagram	4 (17)
MySpace	3 (13)
Vine	2 (8)
Twitter	1 (4)

### Shared Personal Information on Social Media

When asked “What information have you posted or shared on social media?” about the personal information they posted and shared on social media, 54% (13/24) of children reported that they shared their names. Similarly, 50% (12/24) of children reported that they shared videos or pictures of themselves. Table 2 presents the type of personal information reported by the children and the number of children who reported sharing it through social media.

**Table 2:** Personal information shared by children through social media (n=24).

Type of personal information	Children who reported sharing, n (%)
Name	13 (54)
Videos or pictures of self	12 (50)
Birthday	9 (38)
Hobbies	8 (33)
Videos or pictures of family or friends	8 (33)
Hometown	5 (21)

When asked “Do you know who has access to what you post?” 38% (9/24) of children believed that only close family and friends had access to the content they posted on social media. When using social media, 38% (9/24) of children also reported that a family member or friend was sometimes present. When asked “Is your teacher with you when you use social media?” 54% (13/24) of children reported that a teacher was never present when using social media at school, whereas 17% (4/24) of children stated that a teacher was sometimes present. Even more so, when asked who helped them download social media apps, 42% (10/24) of children stated that no one helped them. Table 3 presents the type of person who helped the children download apps (downloading assistant) and the number of children who reported that type of person helped them within this context.

**Table 3:** Children’s reported downloading assistance of social media apps (n=24).

Downloading assistant	Children who reported assistance, n (%)
None	10 (42)
Parent	8 (33)
Other	8 (33)
Sibling	4 (17)
Friend	1 (4)

### Quality of Life

Children who reported that they used social media were also asked to complete the PedsQL portion of the survey to explore their perceived quality of life. Overall, 57% (24/42) of children answered this section of the survey. When reporting on emotional functioning in the context of social media use within the past month, 71% (17/24) of children stated that they never felt sad or blue, 63% (15/24) of children indicated that they never felt afraid or scared, and 54% (13/24) of children reported that they never felt angry. Table 4 presents the emotional functioning items of the survey and the number of children who reported that they never had a problem with each item.

**Table 4:** Children’s reporting on emotional functioning using the Pediatric Quality of Life Inventory (n=24).

Emotional functioning item	Children who reported the item as “never a problem,” n (%)
Feeling sad or blue	17 (70)
Feeling afraid or scared	15 (63)
Feeling angry	13 (54)

When reporting on social functioning in the context of social media use within the past month, 50% (12/24) of children stated that they never had difficulty making new friends, 44% (10/24) of children indicated that they never had difficulty with others wanting to be their friend, 39% (9/24) of children stated that they never had difficulty talking to other children at recess, and 50% (12/24) of children reported that they never had difficulty talking to their parents or siblings. Table 5 presents the social functioning items of the survey and the number of children who reported that they never had a problem with each item.

**Table 5:** Children’s reporting on social functioning using the Pediatric Quality of Life Inventory (n=24).

Social functioning item	Children who reported the item as “never a problem,” n (%)
Difficulty making new friends	12 (50)
Difficulty with others wanting to be their friend	10 (44)
Difficulty talking to others at recess	9 (39)
Difficulty talking to parents or siblings	12 (50)

When reporting on school functioning in the context of social media use within the past month, 30% (7/24) of children reported that they never had difficulty paying attention in class, 44% (10/24) of children stated that they never had difficulty participating in class, and 48% (11/24) of children indicated that they never had difficulty keeping up with schoolwork. Table 6 presents the school functioning items of the survey and the number of children who reported that they never had a problem with each item.

**Table 6:** Children’s reporting on school functioning using the Pediatric Quality of Life Inventory (n=24).

School functioning item	Children who reported the item as “never a problem,” n (%)
Difficulty paying attention in class	7 (30)
Difficulty participating in class	10 (44)
Difficulty keeping up with schoolwork	11 (48)

When reporting on physical functioning in the context of social media use within the past month, 48% (11/24) of children indicated that they never had difficulty being active at recess, 57% (13/24) of children reported that they never had difficulty participating in gym class, 30% (7/24) of children stated that they never had difficulty wanting to go outside and play at home, and 30% (7/24) of children indicated that they never had difficulty wanting to join any sports teams. Table 7 presents the physical functioning items of the survey and the number of children who reported that they never had a problem with each item.

**Table 7:** Children’s reporting on physical functioning using the Pediatric Quality of Life Inventory (n=24).

Physical functioning item	Children who reported the item as “never a problem,” n (%)
Difficulty being active at recess	11 (48)
Difficulty participating in gym class	13 (57)
Difficulty wanting to go outside and play at home	7 (30)
Difficulty wanting to join any sports team	7 (30)

## Discussion

### Principal Findings

#### Overview

Overall, our principal findings suggest that Canadian children aged between 6 and 10 years are active users of social media. By conducting our survey with one-on-one interviews, we found that Canadian children aged between 6 and 10 years use social media, share their personal information on social media, and download apps without consistent supervision. These findings contribute to the existing knowledge gap in the Canadian literature regarding young children’s digital literacy and social media use by incorporating the perspectives of a largely understudied proportion of the Canadian population, which is vital for understanding digital inclusion in Canada.

#### Social Media Use

Approximately half of the children in the study reported that they used social media. This finding is slightly higher than that reported by Canadian researchers [22], who found that approximately 30% of Canadian students had Facebook accounts and 16% had Twitter accounts. Longitudinal studies in the United Kingdom, which explored child and youth engagement with digital technologies, attest to the rapid growth in social media engagement within the past few years [31]. Most notably, parents from the United Kingdom reported that children aged 3-4 years were using the internet at an average of 8 hours per week, children aged 5-7 years were using the internet for approximately 9 hours per week, and children aged 8-11 years were using the internet at an average of 13.5 hours per week [31].

#### Social Media Knowledge

When asked, children in this pilot study described social media as a commercial product by using proprietary names, such as YouTube, Snapchat, and Facebook. The children’s identification of YouTube as a preferred social media site remains consistent with the preferences of 48%, 71%, and 81% of British children aged 3-4, 5-7, and 8-11 years, respectively [31]. YouTube’s platform may optimize fundamental digital literacy skills and is thus relatively easy for children to navigate, search, and interact with. It also offers children multisensory entertainment, learning opportunities from educational videos, and the ability to create their own content. Multisensory learning activities have been suggested to enhance learners’ knowledge and skills with a multisensory experience, including auditory, visual, and kinesthetic stimulation, which may engage and maintain children’s attention for long periods [32].

#### Personal Information Shared on Social Media

What is perhaps most concerning in this study is the nature of personal information and content shared by the children through social media in addition to their lack of awareness regarding who had access to their posted information. Developing evidence indicates that many parents and children are unaware of internet privacy policies and how third parties can collect and use their personal information [33]. Similarly, a large proportion of Canadian youth believe that personal information cannot be collected by commercial corporations if a privacy policy exists [22]. It may also be that children are unaware of the importance of consent when posting content of others and the permanence of their digital traces when trying to delete previously posted content from sites [22]. Although privacy settings on social media sites are purportedly designed to protect users from potential harm, they are more often than not incomprehensible to children and most adults [7]. Nevertheless, it is important to recognize that sharing personal information and self-produced content on social media is a critical part of identity formation for children and, when handled correctly, can strengthen their relationships with others [19].

Parents and teachers of young children play a significant role in the development of children’s digital literacy skills, especially as it relates to personal safety in terms of information sharing, privacy settings, and how to protect oneself from unsolicited or unwanted web-based interactions [22]. The ability of parents and teachers to support the development of digital literacy skills among children is often a limited variable [34,35]. However, the development of effective digital literacy skills among parents is especially relevant, given that young children use digital devices and social media as modeled by their parents [17]. When investigating children’s digital literacy, as it relates to personal information sharing and internet safety, researchers found that many parents tend to use a “do what I say not what I do” strategy, whereby they caution their children regarding internet safety but will engage in unsafe internet behaviors and model excessive device use at home [34].

There are important educational opportunities to increase awareness of role model behaviors in the context of digital literacy and internet safety [34]. Parental behavior, along with the family home environment more generally, are influential factors that socialize and shape children’s habits and, as such, should be optimized to promote best practices when it comes to digital devices and social media use [17]. Furthermore, education policies in Canada created since the beginning of the COVID-19 pandemic promote elementary students’ web-based learning [36,37] and ostensibly increase the influence that teachers have on children’s digital literacy skills. Elementary teachers have an opportunity to integrate digital literacy topics into existing learning activities, such as narrative writing in the first grade, to begin solidifying key concepts at an early age.

Canadian researchers claim that Canadian youth “seek both privacy and publicity at the same time by posting information and then seeking to control the various audiences that can access it” [22]. As Canadian youth seem to approach privacy in digital spaces differently than previous generations, the current Canadian legislation “may not only fail to meet their needs but actually increase the kinds of vulnerabilities they face online” [22]. In terms of web-based supervision at home, approximately one-third of the children active on social media reported that a family member or friend was present while they were on social media, whereas when downloading content from the app store, approximately half of the children reported that no one helped them. This could potentially be because of the understanding that children seek privacy from authoritative figures such as parents while using social media and unknowingly choose self-exploration at the expense of safety [38].

Importantly, unsupervised downloading practices raise concern with respect to the terms and conditions children agree to when they download social media apps, such as the monetary and personal information costs levied on app users to access app content. In granting consent to access the content within popular children’s social media apps, such as those identified by the children in this study, individuals often consent to the app’s collection and sharing of user device information, such as device type identification (eg, smartphone and tablet), device brand identification (eg, Apple and Samsung), device software identification (eg, most up-to-date operating system and previous operating system), device means of connectivity (eg, hardwired internet, wireless modem internet, and wireless cellular internet), and device geolocation to commercialized third parties [33].

The failures of privacy legislation are highlighted in a recent report by the US Federal Trade Commission [33], which documented a significant lack of users’, both parent and child, awareness and concern toward personal information exploitation by third-party commercial enterprises among app users. The report noted that the limited transparency and high digital literacy demands of the privacy policies attached to child-focused social media apps [33]. The existing user agreement and consent jargon make it difficult for most parents to make informed decisions regarding the quality and safety of apps for their children and nearly impossible for a child to comprehend when independently downloading their own apps. There is growing awareness of the educational curriculum regarding the development of digital literacy skills and regulations that safeguard information privacy among social media users.

These findings have further implications for areas of study, such as digital health and, by extension, digital health literacy [39,40]. Digital health accounts for the ways in which digital devices permeate every aspect of daily life, in other words, how digital devices affect our overall well-being. [41] Digital health examines the benefits and drawbacks of various technologies aimed at smartphones, smart objects, social media, wearable technologies, websites, forums, and health-related apps [41]. For example, child health researchers have found that social media serves as a support network for health-related matters and a distraction from chronic illness among older children [5].

To effectively engage with and maximize the health benefits afforded by digital devices, users must have sufficient digital health literacy skills [42]. Digital health literacy, also known as eHealth literacy [43], is defined as “the ability to seek, find, understand, and appraise health information from electronic resources and apply the knowledge gained to addressing or solving a health problem” [44]. Simply put, digital health literacy skills integrate the knowledge of multiple literacies, including traditional (writing, reading, and understanding numeracy), computer (managing data privacy and personal information), media (critically assessing mass media sources), scientific (discerning evidence from opinion), information (searching and retrieving relevant information), and health (comprehending health information and systems) literacies [44]. These intersecting literacies further fall into two skill categories, analytical and context specific, given that some demand more sophisticated skills from a user than others [44]. Traditional, media, and information literacy comprise the analytical skills necessary for a user to critically analyze various sources of information [44]. However, scientific, health, and computer literacy comprise context-specific skills, as they depend on specific issues (eg, discipline terminology), problems (eg, health issues), or contexts (eg, web 2.0) that require nuanced guidance beyond analytical literacies [44].

Considering North America’s information and data-driven society, sufficient digital health literacy skills are, and will continue to be, a crucial component of full and informed participation within evolving educational, civic, corporate, social, and economic spaces [5-7]. It is, therefore, essential that digital device users of all ages, including young children, develop proficient digital health literacy skills to best support their overall well-being [45]. This is especially pertinent given that emerging evidence suggests digital natives—that is, youth who grow up with digital technology—lack adequate digital health literacy skills [46].

#### Quality of Life

Research findings related to social media use and children’s emotional well-being, physical activity, social relationships, and school performance are mixed [47-49]. There is ongoing debate in the literature as to whether children’s and adolescents’ social media use enhances or detracts from their emotional well-being [49,50]. Recent research shows that social media use can be used to support adolescents’ self-esteem [50]. The results of this study are consistent with the results of a previous study [50], as more than half of the children who were active on social media reported being happy. As children’s happiness may be related to their learning [51], future research that examines the relationship between children’s social media use, happiness, and learning outcomes may further elucidate the extent to which social media use and digital literacy skills impact children’s well-being.

Regarding physical activity, nearly half of the children in this study reported that they never had difficulty playing outside at recess or participating in gym, which contrasts with previous findings that screen time is negatively associated with physical activity [47]. Half of the children in this study also claimed they did not have difficulty making friends, which is consistent with findings that social media use contributes to friendship development and support among adolescents [52]. However, limited or no access to social media may create an exclusionary social environment in which some children may face inequitable barriers to social or learning opportunities. Thus, further research should examine the role of social media in creating young children’s social environments and how participation in such environments influences children’s digital literacy skills.

Finally, recent evidence shows that screen time, especially during weekend hours, detracts from good academic performance among adolescents [47]. Additional research is needed to further explore the relationship between screen time and academic performance among very young students, given that almost half of the children in this study reported little to no difficulty with schoolwork completion, which supports previous findings [53]. Furthermore, the transition to web-based learning across Canada in response to the COVID-19 pandemic prompts a reexamination of screen time’s effect on children’s academic performance, as elementary students are attending school through the internet from home and thus learning via screen time. Such research may provide insight into the evolving relationship between children’s screen time, digital literacy, and academic attainment while simultaneously providing information that may advance digital literacy pedagogies developed for elementary students.

### Limitations

This pilot study has several limitations. In this study, data were collected from elementary students involved in a school-based health promotion program within less affluent neighborhoods in Southwestern Ontario. Children from more affluent neighborhoods may report differently on their use of social media. Only children who spoke English as their first language were included in the study; the use of social media apps may be very different for children whose first language is not English. Cultural differences may influence social media use, which was not a focus of this research. Although the children in this study were active and healthy, children with acute or chronic disease or with physical disability may take up social media differently. This pilot study was conducted in an urban after-school program, which was a necessary geographical limitation because of its exploratory nature. The small sample size of this study was not powerful enough to complete statistical analyses beyond descriptive measures. Finally, the cross-sectional design of the study also limited the analysis of children’s social media use over time and is not necessarily a representative sample of this population.

### Future Research

Future research with this population should consider the possible influence that various social determinants of health, such as citizen status (immigrant or refugee), cultural background, disability, geography, and social class, may have on children’s uptake, use, and motivations for engaging with social media. For example, future research that evaluates how children living in a rural community access and use social media is needed to further conceptualize the impact that geographic remoteness and potentially limited access to a reliable internet connection may have on children’s engagement with social media. Social forces such as gender norms should be given further attention, as they may impact a child’s experience with social media at large as well. Future work with this population should also take into consideration the implementation of longitudinal and/or more substantial cross-sectional design, as they can deepen the understanding of children’s relationships with social media and the extent to which this relationship is influenced by social determinants of health, such as age, gender, socioeconomic status, and ethnicity.

This study also has implications for the study of digital health literacy among this population. Although evidence shows youth are engaging with social media, there is limited research seeking to understand the rapidly evolving uptake of, and practices relating to, social media among children aged ≤10 years, which creates a significant gap in knowledge concerning children’s digital health literacy. For example, the context-specific domains of digital health literacy, namely, health and scientific literacy, should also be considered as further areas of study, as each contains the skills needed to understand discipline-specific terminology, execute effective search strategies, and assess health information quality.

In light of COVID-19 and the shift to remote e-learning, research that aims to explore young children’s digital literacy can contribute to evolving models of preschool and elementary e-learning education by examining inequities and supports in relation to children’s engagement and success within a virtual education context. Moreover, examining children’s digital health literacy competency within the age of COVID-19 can provide further insight into how children manage their health during a pandemic across a digital landscape of various social media platforms and information sources, in addition to how they understand the consequences of public health policy, such as contact tracing and social distancing, which has affected the structure of their learning environment.

### Conclusions

Our findings suggest that Canadian children aged between 6 and 10 years are active social media users. This study highlights how the uptake and adaptation of social media use among particular age groups is constantly evolving and worth tracking over time. Many children demonstrated knowledge of the term social media, as they understood it in two distinct ways: by the term itself or by a proprietary name such as Facebook. This raises questions as to where children receive their digital literacy knowledge from and whether such knowledge is gained through self-directed exploration or observation from other actors, such as parents, siblings, or teachers.

The digital literacy practices reported by the children in this study have implications for their health, social and learning behaviors, and safety. Children shared their personal information in addition to self-generated content, such as pictures and videos of themselves and others through social media. This has led to the implementation of early childhood digital health literacy education to best guide, support, and empower children when making these choices to fully understand the social consequences and permanency of their actions. Children’s reported unsupervised social media use, particularly the downloading of apps, reinforces the notion that young children are self-directed users of digital devices and social media. In turn, user acknowledgment and support should be given to this population, as they are just as engaged as older youth populations and perhaps even more vulnerable to web-based threats due to the oversight of privacy policy development thus far in Canada.

## Abbreviations

PedsQL: Pediatric Quality of Life Inventory
